# A systems approach to assessing complexity in health interventions: an effectiveness decay model for integrated community case management

**DOI:** 10.1080/16549716.2020.1794106

**Published:** 2020-08-10

**Authors:** Aliya Karim, Daniel Cobos Munoz, Daniel Mäusezahl, Don de Savigny

**Affiliations:** a Department of Epidemiology & Public Health, Swiss Tropical and Public Health Institute (Swiss TPH), Basel, Switzerland; b Department of Epidemiology & Public Health, University of Basel, Basel, Switzerland

**Keywords:** iCCM, systems thinking tools, bottleneck analysis, sankey diagram, process mapping, data visualization

## Abstract

Complexity is inherent to any system or program. This is especially true of integrated interventions, such as integrated community case management (iCCM). iCCM is a child health strategy designed to provide services through community health workers (CHWs) within hard-to-reach areas of low-and-middle-income countries (LMICs). It is comprised of many interlinked program components, processes and stakeholders. Elucidating the complexity of such programs is essential to designing interventions that respond to local contexts and successfully plan for sustainable integration. A pragmatic approach has yet to be developed that holistically assesses the many dimensions of iCCM or other integrated programs, their alignment with local systems, and how well they provide effective care. We propose an accessible systems approach to both measuring systems effectiveness and assessing its underlying complexity using a combination of systems thinking tools. We propose an effectiveness decay model for iCCM implementation to measure where patient loss occurs along the trajectory of care. The approach uses process mapping to examine critical bottlenecks of iCCM processes, their influence on effectiveness decay, and their integration into local systems; regression analysis and structural equation modeling to determine effects of key indicators on programmatic outcomes; and qualitative analysis with causal loop diagramming to assess stakeholder dynamics and their interactions within the iCCM program. An accurate assessment of the quality, effectiveness, and strength of community-based interventions relies on more than measuring core indicators and program outcomes; it requires an exploration of how its actors and core components interact as part of a system. Our approach produces an interactive iCCM effectiveness decay model to understand patient loss in context, examines key systems issues, and uses a range of systems thinking tools to assess the dynamic interactions that coalesce to produce observed program outcomes.

## Background

### Complexity in community health interventions

Child health interventions in low- and middle-income countries (LMICs), which frequently target children directly at community level, are increasingly complex endeavors. Such programs often entail a combination of services and processes, which are influenced by programmatic inputs, local stakeholders, and their operational setting [1,2]. Integrated Community Case Management (iCCM) is one such program that addresses high child mortality in hard-to-reach areas by training and equipping community health workers (CHWs) to carry out case management within their communities for some of the primary causes of child deaths: pneumonia, malaria, and diarrhea [3–5]. The World Health Organization (WHO) and the United Nations Children’s Fund (UNICEF) advocate iCCM of common childhood illnesses as ‘an essential strategy that can both foster equity and contribute to sustained reduction in child mortality’ [6,7]. Currently, almost all countries in sub-Saharan Africa have adopted some form of iCCM program or policy [8].

The iCCM programs of African countries are generally implemented by local ministries of health (MoH) in partnership with development agencies and non-governmental organizations (NGOs), and are usually supported by the WHO and common international funding bodies [8]. Programs are often multifaceted, and can focus on recruiting, training, and equipping health workers or further developing existing cadres with service capacities and commodities; training supervisors at district and health facility level and ensuring consistent supervision; fostering child health policy change and facilitating implementation; identifying local financing mechanisms; developing ministry-led administrative teams; creating supply chains and data collection pipelines; and mobilizing communities and local leaders. Often, many strains of iCCM are implemented in parallel within countries by different agencies. These numerous components, stakeholders and processes, and their interactions all impart inherent complexity to the intervention.

In order to ensure that such complex community-based programs can be both robust and sustainable, they must be readily adaptable, and integrate fluidly into local systems [9,10]. This is true not only for iCCM, but preventive and curative health approaches across the primary health care (PHC) spectrum. This requires evidence-based evaluations which not only account for complexity, but actively examine how the dynamic nature of intervention components generates observed outcomes [11–15]. Moreover, because policy and program design decisions are usually not made by researchers, such evaluations must be clear and useful to not only an academic audience but local program managers and decision-makers that shape and influence implementation [16,17]. While some studies have addressed different aspects of complexity within iCCM, there is a paucity of literature documenting outcome consequences of differences in iCCM structure, strategy and implementation and how these interface with the local health system [18–20]. Furthermore, while determinants of sustainability of iCCM have received some visibility, how differences in program strategies translate into sustainability has yet to be explored to our knowledge [19,21].

An optimal way to address these questions is to use Systems Thinking, an approach to examining how a system’s components interact with each other and within their respective contexts [22]. More simply, it is a way to understand and intervene in systems. The practice of Systems Thinking is often associated with various ‘tools’ to aid in the analysis of the complexity of a system [23]. In this paper, we build on systems thinking theory to propose an approach to the analysis and evaluation of the iCCM intervention that can also be translated to assessing complexity in general health programs. This research approach applies mixed-methods and different tools to examine how program structure, actors and processes interact with context to influence program effectiveness and its potential sustainability. We intend to bridge the academic and technical spheres of implementation research by proposing an approach that provides pragmatic results useful to implementers while using rigorous systems research methods.

### iCCM as a complex adaptive system

The concepts guiding this methodological approach operate under the domain of Complex Adaptive Systems (CAS). CAS are described as ‘open-ended systems’, which means they are characterized by continuing self-organization and non-linear connections influenced by the interface among their components and actors [24]. The idea underlying CAS is that examining one part of a program or system will not necessarily reveal how that program or system will behave. This reality renders them an appropriate way to approach the complexity within the iCCM intervention.

## Aims & objectives

The aim of this paper is to provide an overview of a comprehensive ‘systems approach’ to analyzing integrated community health programs in LMICs, specifically iCCM. This systems approach is guided by five overarching evaluation domains within the context of iCCM, which are assessed through the application of the approach:

1. How does effectiveness across the trajectory of care change before and after the implementation of the iCCM intervention, and what causes can we attribute to this change?
2. Where do bottlenecks in key systems processes occur, and why?
3. What design, context, demographic, and service delivery factors are determinants of programmatic and health outcomes within the iCCM intervention?
4. How do context and actor interactions affect the implementation of iCCM?
5. What is the extent of systems integration of the intervention, and how does this relate to potential sustainability of iCCM within countries of implementation?

To develop an approach to answer these questions, we built upon systems theory to identify relevant frameworks through which to view the study. This informed study objectives and guided the selection of systems thinking methodological ‘tools’ to achieve them. This culminated in the development of the Systems Approach to the analysis of iCCM, which is comprised of four assessment components and relies upon a combination of systems tools and mixed-methods. In the following sections we describe these frameworks, tools, and the Systems Approach.

## Conceptual frameworks & tools

### Conceptual frameworks

In this study, we use three systems frameworks and three systems thinking tools to guide our analytical approach. These conceptual frameworks and tools were chosen based on their unique properties, their accepted use within systems analyses and the holistic lens that they bring to answering complex questions. Multiple frameworks were required to complement each other to best assess the dynamic nature of the iCCM intervention. Tools were chosen based on not only their methodological rigor, but their practical applicability. To our knowledge, these have not been combined for use in previous studies.

#### Building blocks of health systems

The building blocks of health systems are an established framework for describing the six central components of a health system. This framework is generally considered the gold standard for analyzing health systems elements. These are (i) governance, (ii) information, (iii) financing, (iv) service delivery, (v) human resources, (vi) and medicines and technologies [25]. The core of the framework is people, where it postulates that the actors in the system influence how these components relate with each other. In this study, we use the building blocks to frame the systems components of iCCM for analysis.

#### Mechanisms of effect framework

**Figure 1.**
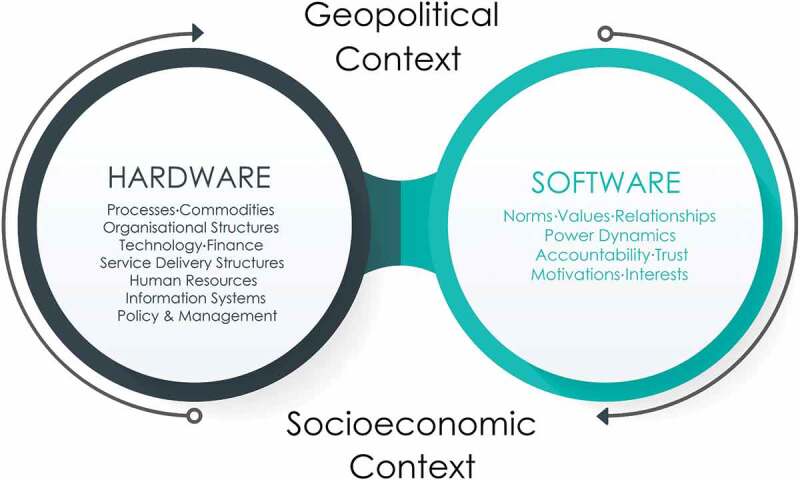
Mechanisms of Effect (Hardware-Software) Framework.

While the Building Blocks of Health Systems offer a wide-angle lens to view the individual components of a health system or program, we also require a framework that qualifies the dimensions of health systems dynamics. The Hardware-Software Framework developed by Sheikh et al. (2011) posits that it is not just the ‘Hardware’ of a system – i.e. its structure, financing schemes, commodities, human resources, service delivery mechanisms, among other tangible factors- but its interplay with the system’s ‘Software’- i.e. the influencing principles and contextual norms, interests, motivations and power flows that shape the relationship of the actors to system elements – which ultimately constitute and determine health system performance [26,27]. We use this concept in our analysis to tease apart the programmatic elements of iCCM and the relationships that underpin them (Figure 1).

#### Design vs. fidelity

We use intervention design and implementation fidelity as an overarching concept throughout the research. Intervention design is simply how an intervention or program and its set of structures and processes is developed to be implemented and function, and fidelity examines or measures its adherence to that intended design [28–30]. The assessment of fidelity has been described as critical to informing evidence-based evaluations and intervention effectiveness [30]. While such assessments can be metrically driven, here we apply this qualitatively to the iCCM intervention to understand how processes deviate from their expected course.

### Systems thinking tools

While these three conceptual frameworks direct our overarching questions and guide the larger ‘picture’ of evidence we aim to gather, we use systems tools to organize and interpret this data. System thinking tools are instruments to help us extract and analyze information about the way a system, organization or program works [23]. We use the following tools and concepts as part of the study approach.

#### Systems effectiveness & effectiveness decay

Health systems effectiveness is a valuable starting point to begin unpacking the complexity of intervention dynamics. While systems effectiveness has been broached in previous work [31–33], it has yet to gain widespread momentum within systems thinking literature or inclusion as a regular part of monitoring and evaluation methods. The concept ascribes the achievement of ‘effective coverage’ of an intervention to the conditional probability of its successive events, beginning with if and where a patient accesses care; whether and how services are administered, received and adhered to; and the success rate of treatment in producing a positive health outcome [34]. It, therefore, takes into consideration both contextual and operational determinants influencing how a patient moves through an intervention, as opposed to a reductionist focus on specific indicators describing only availability, accessibility, or treatment rates in isolation. This is especially valuable within the iCCM context, as the intervention comprises multiple steps in order to achieve an effective outcome.

**Figure 2.**
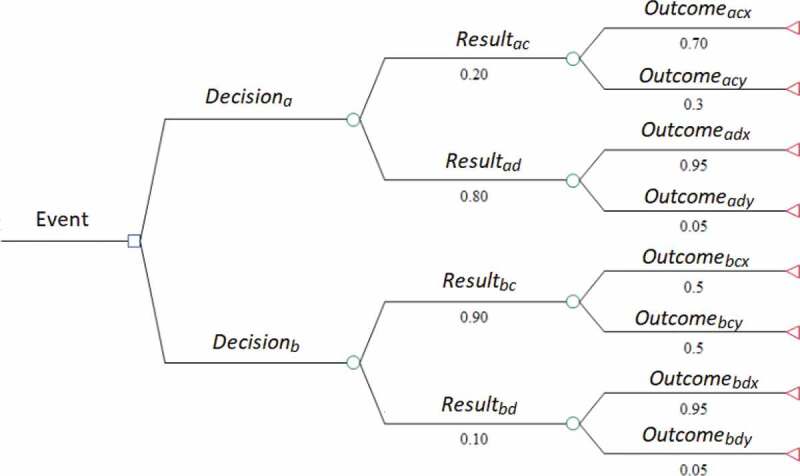
Decision tree model example.

**Figure 3.**
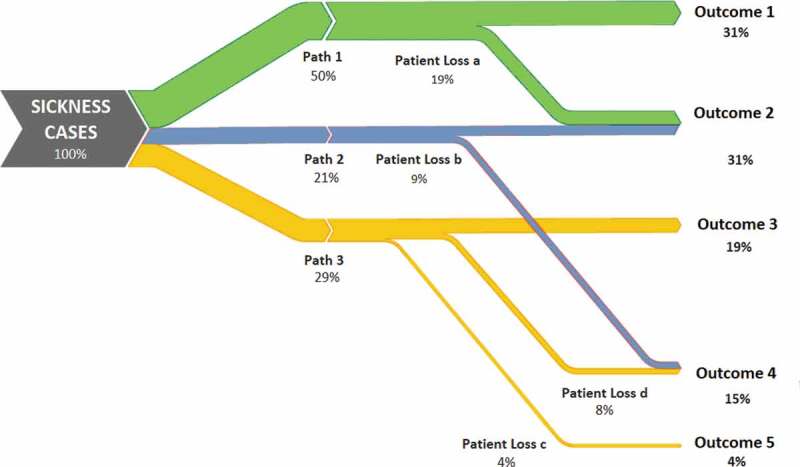
Sankey diagram example.

Effectiveness decay can take a variety of methodological and conceptual forms. Clinically, it has been referred to as the ‘Cascade of Care’, primarily in reference to assessing performance of the care continuum for HIV and certain chronic diseases [35–37]. It is represented econometrically as Decision Tree Modelling (Figure 2), a computation tool to model an algorithm and its series of possible consequences using conditional control statements [38,39]. This is often used in health economics research to perform cost-effectiveness modelling [40,41]. It can also be related in terms of Sankey diagramming (Figure 3), conceptual diagrams of varying band widths to represent flow quantity [42].

Here we conceptualize systems effectiveness in terms of a proposed trajectory of care within the iCCM intervention, and expand upon this construct by describing its ‘decay’ as the loss of cases across this continuum. Effectiveness decay treats each potential area for case loss, or ‘node’, as subject to the mutability of the surrounding health system and as a function of many concurrent forces between its actors, context, and structures.

#### Enterprise architecture & process mapping

**Figure 4.**
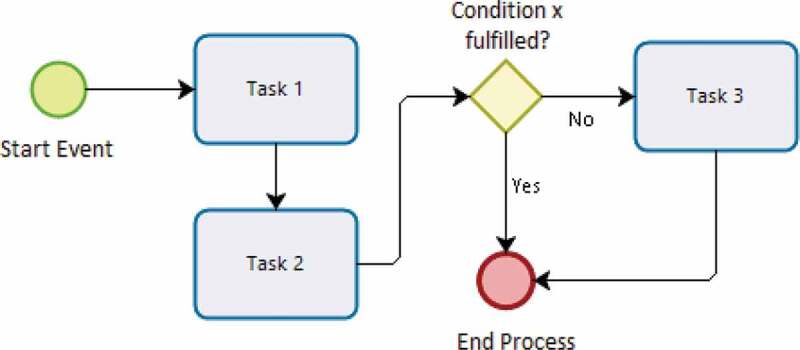
Process map example.

While effectiveness decay models highlight areas of deficiency requiring targeted attention, they do not reveal the underlying causes of such decay, much less expose their interactions with the overarching system. To disentangle this complexity, we use process mapping as a secondary tool. Process mapping and modeling fall within the domain of Enterprise Architecture, a body of methods which aim to describe the fundamental properties of an enterprise or system through the exchange among its elements [43]. The method diagrams the activity flows and actors involved in a process, providing a visual schematic of its possible paths. Process modeling is relatively new in application to the evaluation of health interventions and systems, but has been used to map and evaluate civil registration and vital statistics systems [44]. Figure 4 illustrates an example of a generic process map. This assessment tool examines critical processes that are associated with potential supply- and demand-side determinants underlying the nodes in the effectiveness decay model. These processes can include those from all aspects of the health system and those particularly important to iCCM, from data transmission mechanisms to drug delivery processes, or social mobilization strategies to training and supervision schemes, among others.

Applying process mapping within the context of iCCM can provide stakeholders with a better understanding of the impact of each step in a process as part of a composite whole, especially for processes that have not been previously outlined or are not well understood. This can promote the identification of bottlenecks and associated relevant stakeholders that may have not been considered previously, while encouraging new exchanges among knowledge brokers, program officials, or downstream actors. Compared to other methods, process mapping is also highly interactive, which allows stakeholders to take an active part in the exercise. Process mapping also has the potential to reduce ‘indicator noise’ by identifying the most critical process steps associated with effectiveness decay, and therefore their associated determinants.

#### Causal loop diagramming

**Figure 5.**
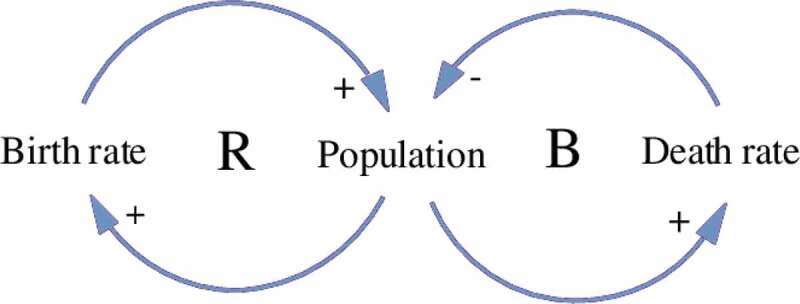
Example of a causal loop diagram modeling population growth.

The final tool we use as part of this Systems Approach is Causal Loop Diagramming (CLD). CLD is an analytical tool to document, model, and visually map the different interactions among a system’s elements, variables or subsystems, and represent the nature and direction of their relationships [23,45–48]. CLD is rooted in the field of systems dynamical modeling, and can be used to build and communicate mental models in a tangible way. Figure 5 is an example of a simple CLD representing determinants of population growth.

In this analysis, causal loop diagrams model and visualize the totality of observed dynamic complexity in iCCM. We use CLDs to illustrate how programmatic aspects of the intervention which affect the critical processes and effectiveness gaps are interrelated with the program’s structure, actors, and context. In other words, CLD is applied to elucidate how iCCM’s hardware and software elements interact and influence programmatic outcomes.

It has been shown that policymakers and managers who better understand the emergent nature of their organizations, programs, and health systems are better able to implement systems-driven solutions [14,49]. CLDs provide an advantage both to assessment of iCCM and the presentation of results in that they capture complexity in dynamic environments, model their movements with analytical rigor, and provide end users such as policymakers and managers with a concrete representation of their system for improved decision-making.

## Methods

### Development of systems approach

We used the conceptual frameworks and tools as guides in the development of the Systems Approach. We first constructed an effectiveness decay model based upon the trajectory of care of iCCM. We defined effectiveness decay within the context of iCCM as case loss as a patient moves through the health system and subsequent intervention. Initial development of the effectiveness decay model was based on the consensus of iCCM technical experts and informed by document review. After construction of the generic model, we performed country-specific contextualization in collaboration with program experts through discussions and technical working groups. This comprised the basis for the first assessment area.

Using enterprise architecture theory, the lens of systems design and fidelity, information from key technical experts, and a program document review, we preliminarily identified processes relevant to iCCM program functioning or important to optimizing its outputs. This formed the foundation for the process mapping component of the approach. The assumption underlying this was that processes would be determined and assessed iteratively, where emergent processes found to be influential to iCCM or its local integration would be incorporated and mapped throughout the systems analysis.

In order to determine the attributable effects of key systems factors on the effectiveness and program outputs within the iCCM intervention, we introduced statistical modeling as a necessary assessment component. We performed a stakeholder mapping exercise of key iCCM actors and used the building blocks framework to determine thematic areas of analysis and their respective indicators and sources.

Finally, the mechanisms of effect framework steered the inclusion of qualitative analysis to understand iCCM actors’ agency, motivations and perceptions. Qualitative data is also necessary to support conclusions drawn from the statistical analysis and the process mapping studies, provide context for areas of effectiveness decay, and determine how these dynamics can potentially affect the sustainability of the intervention over time. Using the framework, we drew upon relevant software and hardware themes within the context of iCCM, and used the stakeholder analysis to determine relevant key actors.

## Results

### Summary of systems approach

The final approach resulted in a combination of mixed methods and systems thinking tools to build a ‘systems picture’ of iCCM to determine where it is losing effectiveness within intervention areas, and what factors, processes and actors interact to influence program outcomes. This research approach comprises four assessment components: (i) We use survey data gathered from before and after program implementation to develop effectiveness decay models across the trajectory of care, measuring changes in careseeking, access and treatment of children after the introduction of the intervention. (ii) We employ process mapping to disentangle processes critical to iCCM activities to understand how they function, where bottlenecks occur, and how these affect effectiveness outcomes. This component also identifies to what extent program processes are integrated into local systems, and their potential to be sustained over time. Data for this are drawn from a document review and key informant interviews (KII) with stakeholders across building block themes and throughout the administrative hierarchy. (iii) To measure which systems factors affect defined program outcomes and to what extent, we perform regression analyses and structural equation modeling (SEM) using survey data from CHWs, supervisors, and caregivers as well as routine monitoring and population data gathered from program records and national statistics. (iv) Finally, to assess actor interactions within the program and with the overall system, we perform qualitative analysis using data on context, motivation, and activities collected from iCCM actors, including supervisors, traditional leaders, CHWs, and caregivers. We use causal loop diagramming to map the direction and feedback loops of these interactions. Table 1 summarizes these assessment components and lists their corresponding data sources.

**Table 1.** Assessment components of the systems approach and their data sources.

Assessment Component No.	Assessment Component	Description	Data Source	Stakeholders
Assessment Component 1	Effectiveness Decay	Measures effective coverage at each stage of the trajectory of care before and after the implementation of iCCM	Household surveys conducted before and after iCCM implementation	Caregivers
			iCCM Routine Monitoring Data	n/a
Assessment Component 2	Process Mapping	Maps key processes within the iCCM intervention, and compares their intended design to adherence to this design	iCCM Program and Policy Documents	n/a
			Key Informant Interviews	District and National Ministry and Program Stakeholders
			Focus Group Discussions	Caregivers, Supervisors, CHWs, Traditional Leaders
Assessment Component 3	Quantitative Analysis	Analyzes programmatic aspects of the intervention, including statistical associations with defined program outputs and health outcomes	Surveys on iCCM activities and perspectives	CHWs, Supervisors
			Household surveys conducted before and after iCCM implementation	Caregivers
			iCCM Routine Monitoring Data	n/a
			Population and Geospatial Data	n/a
Assessment Component 4	Qualitative Analysis	Assess power, agency, accountability, and other dynamics between key stakeholders in iCCM	Focus Group Discussions	Caregivers, Supervisors, CHWs, Traditional Leaders
			Key Informant Interviews	District and National Ministry and Program Stakeholders

### Effectiveness decay model

Our iCCM effectiveness decay model is deconstructed into ten key areas, or nodes, where potential case loss can occur. Entrance into the trajectory of care begins with a child illness case, characterized specifically either by fever, coughing with fast breathing, or diarrhea. To ensure systems effectiveness a caregiver must seek care from an appropriate source; a trained health facility worker or CHW must be available to receive the case; a test must be conducted (in the case of cough and fast breathing or fever); appropriate treatment administered in the case of a positive test result or diarrhea; a referral issued in the presence of danger signs or comorbidities; and the referral adhered to by the caregiver. The model does not calculate the effectiveness of cases which first sought care at a health facility, and rather subsumes them under careseeking at appropriate sources outside of a CHW. Additional File 1 lists these nodes and their respective definitions.

Table 2 describes these nodes categories, their driving operators and indicators, and expected data sources for the effectiveness decay component of the study. Operators are the stakeholders, entities, or phenomena that control the action underlying the node. The implementation indicators listed describe the overarching epidemiological and conceptual measures used to categorize the potential dependencies of the node. For example, the Treatment node, or whether or not a case is appropriately treated, can depend on whether the CHW complied with appropriate treatment algorithms and administered drugs correctly and in appropriate quantities (compliance); whether or not the drugs administered were formulated adequately (drug efficacy); or whether or not drugs were available to be administered (commodity availability). Additional File 1 provides detailed definitions of these nodes.

**Table 2.** Data sources and descriptions for effectiveness decay.

Node Category	Description	Operators	Overarching Implementation Indicator(s)	Data Source
Condition	Entrance to the trajectory of care. Describes the proportion of <5 cases with at least one of three primary symptoms associated with major illnesses of iCCM package: fever (malaria); cough & fast breathing (pneumonia); or diarrhea.	Context	Incidence	Household Surveys with Caregivers
Careseeking	Describes whether or not the caregiver sought care for the child. Careseeking can include both appropriate sources of care.	Caregiver/Recipients	Knowledge & Awareness Availability & Accessibility	Household Surveys with Caregivers
Source of Care	Describes where care was sought for the child. This can be at an appropriate source of care such as a health facility or clinic, the CHW, an authorized pharmacy; or an inappropriate source of care such as a traditional healer, the market, PPMV, or a friend or family member.	Caregiver/Recipients	Acceptability Availability & Accessibility	Household Surveys with Caregivers
Case Seen	For those cases for which care was sought at a CHW, describes whether or not the CHW was available to see the child when the caregiver sought care.	Provider	Availability & Accessibility	Household Surveys with Caregivers
Testing	For those cases which were seen by the CHW and presented either fever of cough and fast breathing, describes whether or not an RDT or rapid breathing test was performed.	Provider Program	Compliance Quality of Care Commodity Availability	Household Surveys with Caregivers Routine Monitoring Systems
Test Result	For fever or fast breathing and cough cases that were appropriately tested by the CHW, describes if the case is positive, negative, or unknown for malaria or pneumonia.	Provider Program	Quality of Care Test efficacy	Household Surveys with Caregivers Routine Monitoring Systems
Treatment	Describes whether or not tested or untested presenting fever, fast breathing and cough, or diarrhea cases were appropriately treated with antimalarials, antibiotics, or oral rehydration salts by the CHW, respectively.	Provider Program	Compliance Quality of Care Drug Efficacy Commodity Availability	Household Surveys with Caregivers Routine Monitoring Systems
Referral	Describes whether or not cases seen, tested or untested, treated or untreated were referred to a referral health facility.	Provider	Compliance Quality of Care	Household Surveys with Caregivers Routine Monitoring Systems
Referral Adherence	Describes whether or not cases which were referred adhered to referral.	Caregiver/Recipients	Compliance Accessibility	Household Surveys with Caregivers Routine Monitoring Systems

Data to populate the nodes is extracted from two sources. Household surveys with caregivers conducted before and after program implementation generate the primary metrics for each node from careseeking to referral adherence. These are compared to service-side reported routine monitoring data collected by either ministries or program partners as a secondary data source. Because routine monitoring data only captures those cases which achieved successful contact with a CHW until referral adherence, this data source is used primarily for triangulation purposes.

**Figure 6.**
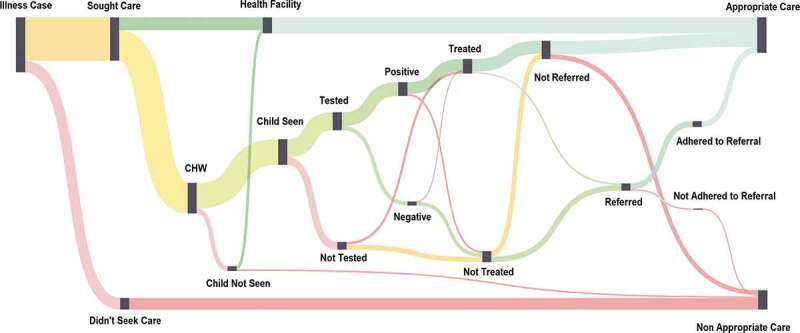
Generic model of systems effectiveness decay of iCCM^†^.

^†^*Figure is condensed for illustrative purposes. Node titles and flows are shortened for brevity. Expanded node nomenclature and definitions are available in Additional File 1*

We analyze the change from before to after the implementation of iCCM for each node by performing Pearson’s Chi-squared test. We develop interactive Sankey Diagrams to visualize effectiveness decay for each country site using Tableau software [50]. These represent cases lost before and after program implementation for each illness condition (fever, coughing with fast breathing, diarrhea) through to whether the case was appropriately treated and/or referral adhered to. Each diagram can be drilled down by node or indicator, and each flow segment or group of patients can be highlighted according to the particular path they took. The number of cases and the percent of the total cases that that group represents can as well be visualized by hovering over any data element in the diagram. Figure 6 represents the generic model for effectiveness decay for iCCM. Definitions for the nodes are available in Additional File 1.

Populated with data, the model can be used to evaluate the conditional probability of a case receiving appropriate care effectively within the iCCM service package. The diagrams are useful to address the ‘denominator problem’, a simple data interpretation challenge imposed by the rigidity of fixed or non-informative denominators, by interactively allowing the viewer to visually understand proportions in terms of a specified variable that they define [51]. Rather than examining a table of all possible combinations of indicator formulations, the viewer has the power to determine the numerators and denominators of their choosing and immediately visualize them as part of a whole. For example, using the diagram the user can decide if they want to view untreated cases as a proportion of cases that were tested, or those that were seen by the CHW, or those that sought care. This allows the viewer to put into perspective relative effectiveness loss, which can be more intuitive and useful in informing program strategy both operationally and financially. Such interactive visualizations are particularly useful to information dissemination in non-academic audiences, who may require a relatively intuitive method of obtaining information rapidly to make informed decisions. This includes program managers or policymakers, who have been found to rely more on mental models of their understanding for decision-making than on written reports or numerical databases [52].

### Process mapping

Processes flagged for mapping were those critical to effectiveness decay or generally considered important within the iCCM intervention. They include those driving supply chains of iCCM commodities, supervision, data transmission, community mobilization procedures, and referral adherence, among others. Data for the process mapping assessment component were drawn from a desk review of program documents, focus group discussions (FGD) with downstream actors such as CHWs and supervisors, and KIIs with program stakeholders. Data from these sources help (i) identify which critical processes are necessary for mapping; (ii) outline the expected design of these processes; (iii) populate process steps with real-time information for comparison of adherence to this design; and (iv) determine to what extent these processes are integrated into local systems and how these processes will be sustained over time. We use Bizagi software to map these processes [53].

### Quantitative analysis

We used two multivariate analyses techniques: structural equation modeling (SEM), and multivariate linear and logistic regression analysis. SEM encompasses a body of modeling methods, which are designed to determine structural relationships between measured variables and latent constructs [54]. The flexibility of this method is especially useful for iCCM, as it can measure the potential impact of underlying systems phenomena that cannot be collected by usual means, particularly the software elements described. We use STATA SE15 software to perform the analysis.

The variables used for quantitative analysis are driven by the results of the effectiveness decay and process mapping assessment components, where analysis is performed iteratively alongside the qualitative assessment. The quantitative analysis component can therefore encompasses many different variables and subsequently produce a variety of models, depending upon the associations of interest.

Endogenous variables are key programmatic outcomes, for example, drugs stocked-in at the community level, community contributions to CHWs, or supervision visits conducted. These can also be treated as exogenous factors in their association to indicators of effectiveness decay, such as cases treated or referral adherence rates. Other covariates used in the analyses span the building blocks, and include demographic information of CHWs and supervisors, data on their activities and services, caregiver behavior, and geographic data.

### Qualitative analysis

Data for the qualitative component are derived from FGDs with lower-level stakeholders, such as caregivers, supervisors, and CHWs on their perspectives of their activities, roles, and experiences within the iCCM intervention. Discussions with traditional leaders and health committee members are also used to gauge community perspectives on social mobilization, support of CHWs, the extent of integration of iCCM within communities, and commitment to sustaining iCCM services. We use KIIs conducted with district and national actors relevant to iCCM as well as a desk review to compare and supplement reported results.

We use the Framework Method as the analytical approach to deductively-inductively perform a coding analysis of transcripts [55]. We triangulate qualitative and quantitative data to develop Causal Loop Diagrams (CLDs) to represent how hardware and software elements critical to iCCM affect each other. We use MaxQDA software to conduct the analysis, and Vensim software to develop CLDs [56,57].

### A unified systems approach

**Figure 7.**
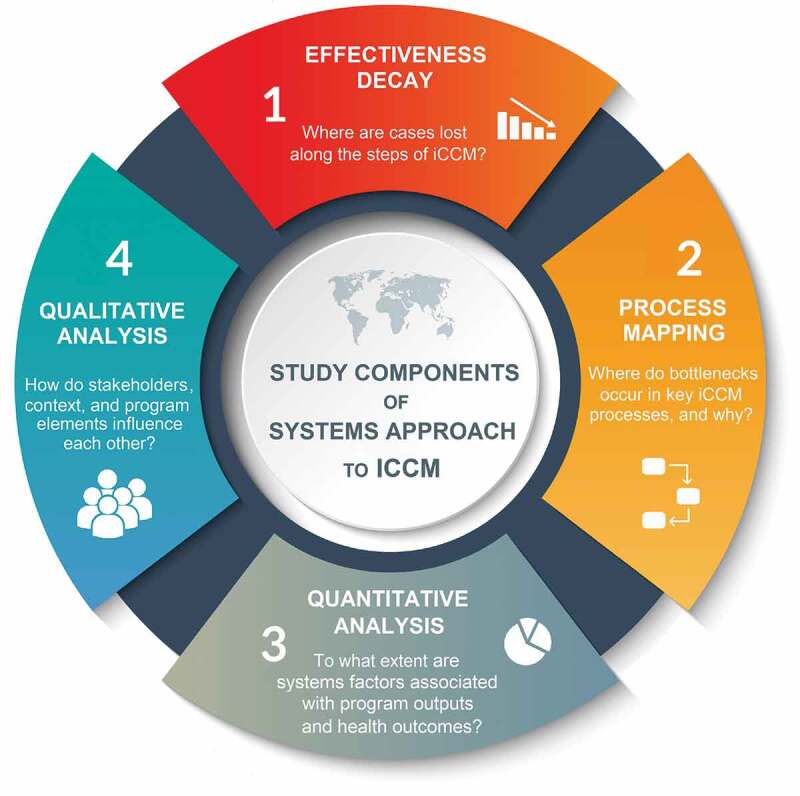
Assessment components of the systems approach of iCCM.

When performed sequentially or iteratively, these four Assessment Components combine to form an analysis approach that is capable of demystifying the complexity of health programs in dynamic settings. This can be applied to health services and systems alike. Effectiveness decay sets the scene to understand where effective coverage is lost throughout the key steps of the service, program or system. This is followed by interviews with key system stakeholders, where process mapping exercises can assist in understanding the complicated nature of the processes affecting this loss in effectiveness. Statistical modelling is then used to concretely assess associations between critical programmatic components that affect these processes and effectiveness. Qualitative analysis supports this by allowing the researcher to explore how and why these phenomena occur. The application of causal loop diagramming links these elements in a dynamic picture that acts as both an academic product of these models and a vehicle for knowledge translation to decision-makers. Figure 7 provides a summary of the overall systems approach.

## Application of approach

In the following section, we describe a practical application of this systems approach within the iCCM context. We summarize the steps involved with the preparation and data collection processes for the four assessment components, as well as their analysis and how they fit together as a whole. Specific protocols for data collection can be found elsewhere [58].

### Instruments & data collection

The study was undertaken in iCCM programs implemented in four large territories of three sub-Saharan African countries. Our team drafted protocols, data collection tools and training materials, specifically survey instruments for CHWs and supervisors; FGD guides for CHWs, supervisors and caregivers; and KII templates for identified systems-level and program stakeholders. These instruments varied according to country context and were guided by applicable aspects of the systems building blocks. These posed questions relating to participants’ demographics and background; catchment area information; service delivery activities; data transmission and reporting; training and supervision; health technology and supply chain performance and responsibilities; experiences with communities, social mobilization, knowledge, careseeking, gender; themes surrounding role, perceptions and motivation; and personal opinions. Key informants were stakeholders at different levels of the administrative hierarchy, and included informants on different parts of the building blocks, where questions focused on their roles, experiences, and observations.

We obtained secondary data on caregivers’ experiences of careseeking and treatment of sick children before (baseline) and after the implementation of iCCM (endline). We collected routine monitoring data from program records and the national health information system. These data provided information on supervision and reporting rates, epidemiological data, testing and treatment information, as well as referrals, adherence and follow-up. National population statistics were obtained from national statistics offices directly or via the NGO. We also collected a variety of national and local child health documents and program documents relevant to the regulation, implementation, and monitoring of iCCM across countries. These documents were provided either by local ministries or the implementing NGO.

### Analysis and unification of assessment components

We used household survey data on experiences of careseeking, testing, treatment and referral for ill children under five to populate the Sankey diagrams and perform effectiveness decay analyses across all four country sites in fulfillment of our first study question. Case loss at each step along the trajectory of care was extracted at baseline and endline. Pearson’s chi-squared test was used to determine the magnitude of change from before to after implementation of iCCM. The representation of data in the form of interactive Sankey diagrams allowed users to toggle through nodes and denominators to determine where effectiveness loss was most acute and along which paths. This case loss set the scene to assess which steps in the care trajectory had been most impacted by the introduction of iCCM, and targeted where effectiveness had the greatest potential to be improved along the continuum.

For each step of case loss, we tagged relevant key indicators and mapped critical processes associated with these nodes using data from interviews with key informants and the document review. For example, at the ‘Treatment’ node, we mapped the supply chain processes of commodities from the international level to the community level and their associated indicators to assess where bottlenecks occurred, and how this may have affected a CHW’s ability to treat. The selection of processes for mapping was guided by the effectiveness decay, the major systems areas of the building blocks framework, and information extracted from KIIs. We used the design-fidelity framework to compare the design and intent of processes with their actual execution, and if shortcomings in outputs were related to the former or the latter. These steps were used to fulfill our second evaluation domain.

We drew upon the surveys with supervisors, CHWs and caregivers, routine monitoring, population, and geographic data to measure associations of key systems indicators with defined outcomes. This analysis component addressed our third evaluation domain. The primary vehicle of analysis was logistic and linear regression modeling. The analyses performed were similarly guided by the building block areas, and also examined emergent questions that surfaced as a result of the previous exercises. For example, we assessed what factors were associated with timely and correct reporting (Data and Information Systems); frequent and timely supervision (Human Resources); careseeking and community support (Community & Social Mobilization); and stockouts within communities and health facilities (Supply Chain & Commodities), among others. Many of these models overlapped building block areas. For example, assessing the association of the use of an mHealth device and drug stockouts fell in both the Supply Chain and Information Systems Building Blocks. Additionally, multiple models were drawn for analogous outcomes.

SEM was a useful statistical tool for evaluating phenomena described in the Mechanisms of Effect Framework. We used SEM to assess how latent constructs of accountability, relationships, and perceptions of support of the CHW interacted with structural program elements and context to influence whether or not volunteer CHWs desired to continue practicing iCCM.

We analyzed qualitative data from FGDs held with CHWs, supervisors and caregivers using grounded theory to explore perceptions and motivations of these stakeholders, and provide context to the effectiveness decay, process mapping, and quantitative analysis. The Mechanisms of Effect Framework was used to guide this component of the systems assessment which addressed our fourth evaluation domain, and focused primarily on the interplay between hardware and software elements. Themes within this assessment component ranged from assessing CHW motivation; factors affecting careseeking and referral adherence; relationships between actors and their effect on the ability to perform services; attitudes of CHWs and supervisors and obstacles to performing their roles; gender, accountability, power, and prestige; social support and the impact of health committees; among others. Emergent themes also guided the covariates analyzed in statistical analysis.

In the final stages, we drew causal loop diagrams to bring together the relationships that emerged during the previous analyses phases. These CLDs were tailored according to specific overarching outcomes that were noted as significant or critical during the four stages of analysis, where each critical outcome formed the central theme of the CLD. These were often the steps in the trajectory of care (such as ‘Appropriate Testing’ or ‘Referral Adherence’), or other major programmatic components (such as ‘Effective and Timely Supervision’ or ‘Regular Availability of Commodities’). Factors found to impact this critical outcome were drawn and grouped according to thematic area or building block.

While not an explicit part of the systems approach, we used resulting data to fulfill our final evaluation domain. we used KIIs and analysis findings to examine to what extent the program was prepared to be sustained beyond program handover, and how well-integrated facets of the program were into local systems.

Process mapping, quantitative and qualitative analysis occurred iteratively. For example, if regression analysis would reveal an association between a process-driven program element and a critical outcome, such as supervision on continuous drug stock levels, this would lead to mapping of supervision schemes and a deeper examination of both the statistical and qualitative data available on this program element. As processes were mapped, qualitative data from interviews and discussions were used to assess discrepancies in adherence to process design and their underlying reasons, while statistical models were used to measure the extent to which certain factors affected outcomes relevant to these processes.

## Discussion

The proposed systems approach provides a novel way to assess dynamic complexity within community health interventions through a combination of systems thinking frameworks, concepts and tools. Specifically, our approach allows for a robust, systems-based assessment of community programs which account for complexity within different components of the system, among different actors, and at different levels. This approach provides an addition to the growing body of literature of systems approaches to the analyses of health programs [59–64]. While the concepts and tools that comprise this approach have been applied singularly in the assessment of health systems and programs, this is the first approach to our knowledge that unites their strengths cohesively. Our application of the approach to iCCM proved successful in that it provided rich insights on many aspects of the program, how its elements interacted with each other, and how it fit within the overall health system.

However, the approach introduces some limitations. It relies on a number of quantitative and qualitative data elements from a variety of sources, which can increase time investment in both the data collection and analysis process, while stretching budgetary limitations. It also introduces the potential for dependency on secondary data sources such as those for routine monitoring data or population statistics, which may not always be available or reliable. There is the possibility for the researcher to find themselves with ‘data densification’ or saturation, where more data is collected than can be processed. This is especially the case in systems assessments, where often many data components at different levels and sectors of the system are required to produce a robust evaluation.

The building blocks framework can pose rigidity in the assessment of systems components, by either emphasizing domains that are not relevant or omitting elements altogether. We attempted to overcome any shortcomings this posed by developing an iCCM Systems Framework in parallel to the systems approach. This framework and its development is detailed elsewhere [65].

Unifying the four steps to provide the full ‘systems picture’ can be done sequentially or iteratively where steps are performed in parallel and built upon simultaneously. While the latter approach was successfully taken in our assessment of iCCM, this may introduce barriers in the analytical process for other contexts, such as knowing when to ‘stop’ iterations of the data-building process, where saturation has been achieved. This can also introduce limitations in a practical sense, especially if assessment components are divided into work packages that are to be submitted sequentially. Finally, for application in non-iCCM contexts, parts of this approach may be less or non-applicable.

## Conclusion

Complex interventions require evaluations that are both comprehensive in approach and holistic in scope. Moreover, they should have the ability to be absorbed and maintained by the local health system. The multi-tiered design of the proposed mixed-methods approach to analyzing iCCM programs and complex community interventions aims to disentangle the many elements that comprise it, while capturing the extent of its integration into local systems, the breadth of experiences of its actors, and their influence on program effectiveness and potential sustainability. This approach is applicable not only to vertical programs such as iCCM, but to the design and assessment of general health services and interventions. Using a range of systems-based tools and interactive techniques, we hope to provide policymakers and program stakeholders at all levels an approach to obtain meaningful evidence that can be more easily translated into effective action.

## Paper context

Methods to assess complexity in health interventions are rarely concrete. Moreover, they come with the added difficulty of translating information so that it is useable by non-academic audiences. We propose a practical systems-based approach to assessing a child health intervention that can be applied to the assessment of other health services or programs. The approach results in useable outputs and interactive data visualization that can better facilitate knowledge translation among decision-makers.
